# Imaging diagnosis and sequential conservative treatment of pediatric traumatic duodenal intraluminal hematoma

**DOI:** 10.3389/fped.2026.1710734

**Published:** 2026-03-31

**Authors:** Xiaoyan Weng, Qianqin Zhou, Zhaofan Qin, Gang Wen

**Affiliations:** 1 Ultrasound Department of Women and Children’s Hospital Affiliated to Ningbo University, Zhejiang, China; 2 Neonatal Monitoring Center of Women and Children’s Hospital Affiliated to Ningbo University, Zhejiang, China; 3 Imaging Department of Women and Children’s Hospital Affiliated to Ningbo University, Zhejiang, China; 4 Department of Pediatric Surgery, Affiliated Women and Children’s Hospital of Ningbo University, Zhejiang, China

**Keywords:** children, conservative treatment, duodenal hematoma, intestinal obstruction, ultrasonography

## Abstract

**Aim:**

To explore the imaging, diagnosis, and treatment of traumatic duodenal intramural hematoma in children, improve diagnostic accuracy, and shorten hospitalization time.

**Methods:**

A retrospective analysis of five children with traumatic duodenal intramural hematoma.

**Results:**

One child had a descending duodenal wall hematoma, two had horizontal wall hematomas, one had an ascending wall hematoma, and one case involving both the descending and ascending parts. All five children were initially diagnosed by computed tomography (CT). The typical images showed “ice melting sign” and “fissure sign,” with no lesion enhancement. Magnetic resonance imaging (MRI) in two children on follow-up showed spindle-shaped or quasi-circular intestinal walls protruding towards the intestinal lumen, local intestinal lumen stenosis, high signal intensity on T1W1 and uneven high signal intensity on T2W1, with no enhancement of the lesion. All five patients underwent multiple dynamic follow-up examinations using ultrasound, which clearly displayed the structure of the intestinal wall, absorption of hematoma, and the degree of intestinal obstruction.

**Conclusion:**

Conservative treatment, such as gastrointestinal decompression and intravenous nutrition, for pediatric duodenal wall hematoma has a good prognosis results in favorable outcomes. Initial CT diagnosis combined with multiple ultrasound follow-up has high specificity for the diagnosis and treatment of this disease.

## Summary

Traumatic duodenal wall hematoma is uncommon in children and the diagnosis is challenging. Our case series showed the value of ultrasound imaging for preliminary diagnosis and follow-up, allowing observation of hematoma resolution and resumption of gut peristalsis. Initial CT diagnosis combined with multiple follow-up ultrasound is the best imaging plan for traumatic duodenal intramural hematoma in children.

## 1 Introduction

Duodenal injury is a severe form of abdominal trauma that accounts for approximately 3% - 5% of all abdominal injuries (1). Traumatic intramural duodenal hematomas are rare in children. Owing to the inability of most pediatric patients to provide accurate medical histories or describe their clinical symptoms, missed diagnoses, misdiagnoses, and delayed treatments often occur. In adults, the diagnosis and follow-up monitoring of duodenal injuries primarily rely on imaging modalities, such as computed tomography (CT), upper gastrointestinal contrast studies, and magnetic resonance imaging (MRI). In pediatric populations, ultrasonography (particularly high-frequency ultrasound) offers unique advantages for both diagnosis and follow-up monitoring (2). This article presents the diagnostic and therapeutic follow-up monitoring of five children with traumatic duodenal intramural hematoma treated at our institution, highlighting the specificity of initial CT diagnosis combined with multiple ultrasounds follow ups in diagnosing and monitoring this condition.

## 2 Methods

### 2.1 Participants

This was a retrospective study. We identified five children with duodenal intramural hematoma who underwent ultrasound diagnosis, follow-up, and complete data collection between May 2022 and June 2024 at the Women's and Children's Hospital affiliated with Ningbo University. The data collected included sex, age, history of trauma, disease course, symptoms, laboratory test results, imaging examinations, treatment processes, and outcomes.

### 2.2 Examination method

All five patients underwent plain and enhanced upper abdominal CT using a Philips Incisive machine and the contrast agent iodixanol. Two patients underwent plain MRI and enhanced scanning using a Philips Achieva 1.5T system and the contrast agent iodixanol. In addition, all patients underwent multiple abdominal ultrasound examinations using a Philips EPIQ7 ultrasound diagnostic instrument with a 2–9 MHz convex array probe and a 5–12 MHz linear array probe, and a GE Voluson E8 ultrasound diagnostic instrument with a 5–8 MHz convex array probe and a 5–12 MHz linear array probe.

### 2.3 Examination content

The location, size, shape, internal structure, and relationship between hematoma and surrounding tissues was examined. All content was reviewed separately by two pediatric radiologists and ultrasound diagnostic physicians with over five years of work experience. In case of any disagreement, a consensus was reached after discussion. This study was approved by the Ethics Committee of our institution (Approval NO.: NBFE-2025-KY-060), who waived the need for individual patient informed consent because of the retrospective nature of the study.

## 3 Results

### 3.1 General information

Among the five children with duodenal intramural hematoma, four were male and one was female, aged 5–10 years, with a median age of 7.2 years. Before the onset of the disease, all five patients had a clear history of abdominal trauma; two cases of injury from fighting, one case of injury from collision with a table corner, one case of handlebar injury, and one case of injury from a car accident. The patients visited our hospital for treatment 0–4 days after their injury. The primary clinical manifestations included nausea, vomiting, and upper abdominal pain. Physical examination revealed tenderness in the upper abdomen but no muscle tension, bloating, or masses. Laboratory examinations revealed elevated white blood cell count in one patient, decreased red blood cell count in three patients, elevated blood amylase levels in three patients, and elevated urine amylase levels in three patients. At discharge, these abnormal findings had all returned to the normal range. The characteristics of the five children are presented in Table 1.

**Table 1:** Presentation of five cases of duodenal intramural hematoma.

CASE	Sex	Age (y)	History of trauma	Visit time	Symptoms	Physical signs	Laboratory Examinations
1	M	10	Handlebar injury	4 days later	Nausea, vomiting, abdominal pain	Upper abdominal tenderness	Elevated white blood cell count, decreased red blood cell count, elevated blood amylase, elevated urinary amylase
2	M	7	Car accident injury	10 h later	Vomiting, abdominal pain	Upper abdominal tenderness	Decreased red blood cell count, elevated blood amylase, elevated urinary amylase
3	M	5	Table corner collision injury	The next day	Vomiting, abdominal pain	Upper abdominal tenderness	Decreased red blood cell count
4	F	6	Fighting injuries	the same day	Abdominal pain	/	/
5	M	6	Fighting injuries	the same day	Abdominal pain	/	/

M, male; F, female.

### 3.2 Imaging data

#### 3.2.1 CT imaging

All five cases with duodenal wall hematoma were imaged by CT before or on the day of admission, including one case involving the descending part, two cases in the horizontal part, one case in the ascending part, and one case involving both the descending and ascending parts. With plain scans, all five cases showed clear boundaries, circular or sausage-like masses protruding into the intestinal cavity to varying degrees, a continuous mucosal layer, and no ulceration or interruption shadow. Two cases showed low density (small hematoma), three cases showed slightly high or high density (with a large hematoma), and during the absorption period, uneven and mixed densities were the main features. Before discharge, follow-up revealed a low density. Two cases showed the “ice melting sign” and one case showed the “fissure sign.” Enhanced scanning showed no significant enhancement of the lesion, and the three cases with large hematomas showed varying degrees of proximal and gastric cavity dilation, suggesting incomplete intestinal obstruction. In addition, one case was accompanied by pancreatic head injury and one case was accompanied by left lobe liver contusion and laceration. Two patients had inferior vena cava compression and three had superior mesenteric artery and vein compression and displacement. Four patients exhibited varying degrees of abdominal fluid accumulation. One patient showed no abnormalities on abdominal CT 30 min after the trauma, but 10 h later, the abdominal pain worsened with vomiting. CT re-examination revealed a hematoma between the wall of the descending duodenum, suggesting that the hematoma did not form immediately significantly after trauma.

#### 3.2.2 MRI

The two patients with MRI conducted during follow-up showed subacute absorption of a hematoma following conservative treatment. The plain scans showing a localized spindle-shaped or quasi-circular intestinal wall in the duodenal area, protruding towards the intestinal lumen, local intestinal lumen stenosis, smooth and continuous mucosal surface, high signal intensity in T1W1 images, and uneven high signal intensity in T2W1 images. Enhanced scans showed no significant enhancement in the lesion, but mild linear enhancement was observed in the surrounding mesentery, mostly due to inflammatory reactions. In addition, one patient had mild pancreatic edema, pancreatic duct dilation, gallbladder enlargement, and intrahepatic and extrahepatic bile duct dilation. One patient had local contusion and laceration of the left lobe of the liver.

#### 3.2.3 Ultrasound examination

All five cases were also examined by ultrasound on the day of admission and after admission. Ultrasound imaging showed circular, sausage-shaped, or elongated oval masses with long axes, consistent with the course of the duodenum. High-frequency ultrasound clearly displayed the structure of each layer of the intestinal wall, indicating that the hematomas were located within the intestinal wall (for four patients between the muscular and mucosal layers, and for one patient between the muscular and serosal layers). The internal echo was mainly a heterogeneous mixed echo and color Doppler showed no blood flow inside the hematomas. Scattered blood-flow signals were observed in the surrounding intestinal walls. High-frequency ultrasound real-time dynamic scanning can clearly demonstrate intestinal peristalsis, the passage of liquid or other contents in the intestinal lumen, and better evaluate the degree of intestinal obstruction, providing guidance for extubation and adjustment of fluid intake and diet. Three cases showed elevation of the superior mesenteric artery (SMA) and compression of the abdominal aorta (AO). In addition, three patients had mechanical intestinal obstruction caused by hematoma compression; one patient had compression of the head of the pancreas, resulting in pancreatic edema, dilation of the bile ducts and pancreatic ducts, and enlargement of the gallbladder. One case was complicated by a local contusion and laceration of the left lobe of the liver, pancreatic injury, and local ascites. All five patients underwent multiple follow-up ultrasound examinations (Table 2).

**Table 2:** Ultrasound diagnosis and follow-up data.

Case	Location	Time of ultrasound examination	Size (mm)	Shape	Internal echo	CDFI	Adjacent
1	Descending and horizontal duodenum	Day 1	54 × 23	Oblong	Heterogeneous echo	No blood flow inside, scattered blood on the surrounding intestinal wall	SMA is elevated, AO is compressed, intestinal obstruction, pancreatic head compression
		Day 5	91 × 50	Sausage-shaped	Heterogeneous echo with partial cystic echo		
		Day 10	60 × 31	Quasi-circular object	cystic hypoechoic		
		Day 20	28 × 13	Quasi-circular object	Hypoechoic		
		One week after discharge	20 × 12	Quasi-circular object	Hypoechoic		
2	Descending duodenum	Day 1	59 × 34	Quasi-circular object	Heterogeneous echo	No blood flow inside, scattered blood on the surrounding intestinal wall	SMA is elevated, AO is compressed, intestinal obstruction, pancreatic injury, left lobe liver contusion and laceration
		Day 5	47 × 21	Quasi-circular object	Heterogeneous echo		
		Day 12	29 × 12	Quasi-circular object	Cystic hypoechoic		
		Day 20	25 × 10	Quasi-circular object	Hypoechoic		
		One week after discharge	Local thickening of intestinal wall	Quasi-circular object	Hypoechoic		
3	Ascending duodenum	Day 1	28 × 14	Quasi-circular object	Hypoechoic	No blood flow inside, scattered blood on the surrounding intestinal wall	/
		Day 3	16 × 7	Quasi-circular object	Hypoechoic		
		Day 5	10 × 5	Quasi-circular object	Hypoechoic		
4	Horizontal duodenum	Day 1	46 × 24	Quasi-circular object	Heterogeneous echo	No blood flow inside, scattered blood on the surrounding intestinal wall	SMA is elevated, AO is compressed
		Day 3	40 × 18	Quasi-circular object	Heterogeneous echo with partial cystic echo		
		Day 7	23 × 12	Quasi-circular object	Hypoechoic		
		One week after discharge	Local thickening of intestinal wall	/	/	/	
5	Horizontal duodenum	Day 1	37 × 25	Quasi-circular object	Heterogeneous echo	No blood flow inside, scattered blood on the surrounding intestinal wall	/
		Day 3	30 × 14	Quasi-circular object	Heterogeneous echo with partial cystic echo		
		Day 7	21 × 10	Quasi-circular object	Hypoechoic		
		Day 10	10 × 5	Quasi-circular object	Hypoechoic		

AO, abdominal aorta; CFDI, color flow Doppler imaging; SMA, superior mesenteric artery.

### 3.3 Treatment plan

After diagnosis, all five children were immediately admitted to the hospital and received conservative treatments such as fasting, gastrointestinal decompression, anti-infection medication, snake venom thrombin to stop bleeding, and intravenous nutritional support. After one week the gastrointestinal decompression tube was clamped and a small amount of water was given orally. For the four patients, dynamic observation of high-frequency ultrasound showed successful passage through the narrow lumen. However, for one patient, real-time dynamic observation showed obstruction. After the second week for this patient, the same ultrasound monitoring method showed that the drinking water passed through the obstructed area. This monitoring approach greatly improves the success rate of extubation and shortens the fasting and average hospitalization times.

### 3.4 Follow-up and prognosis

**Figure 1:**
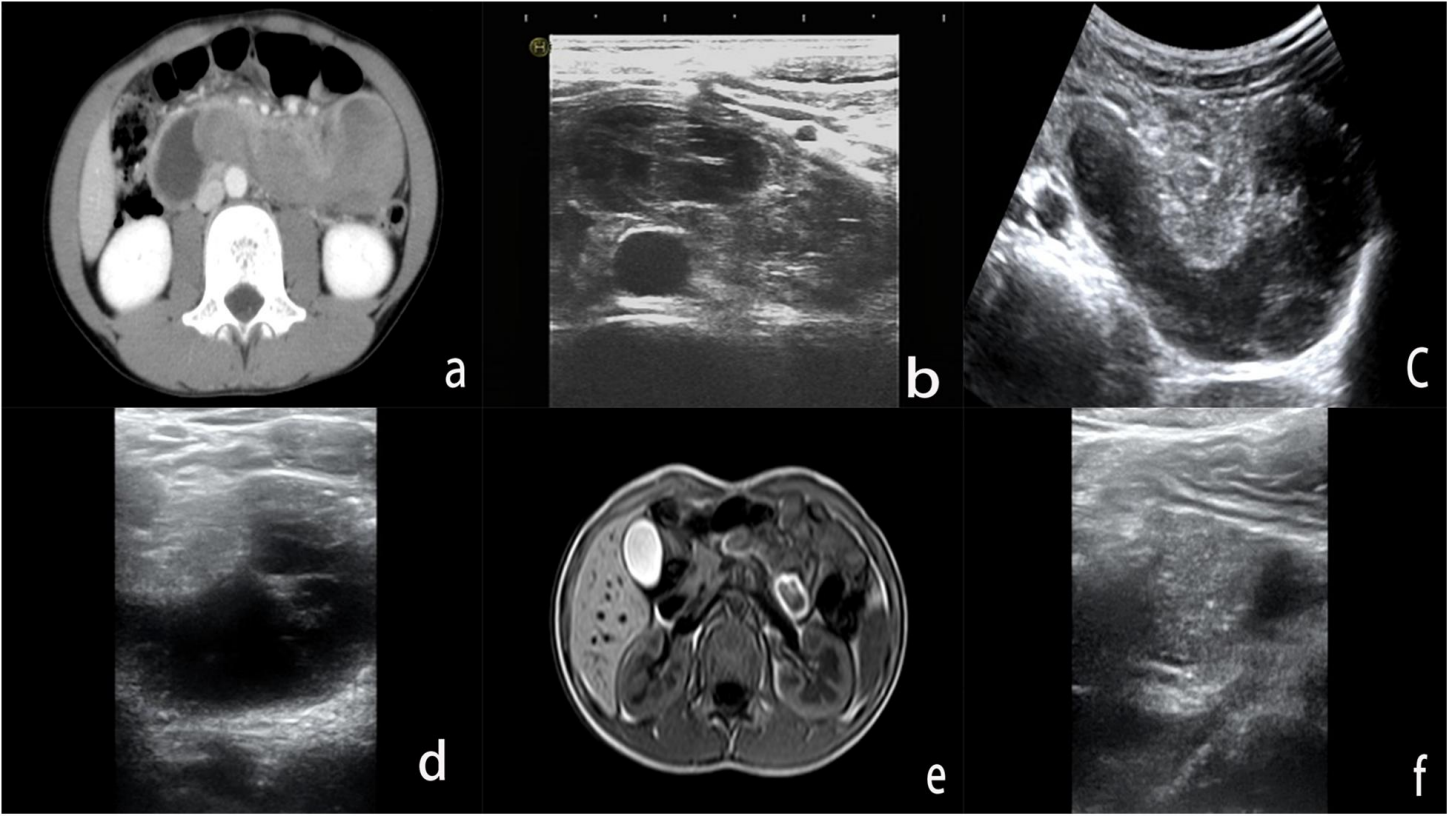
Imaging examination of duodenal wall hematoma in a child with car injury. **(a)** On the day of injury, CT scan showed a long, clustered, slightly high-density shadow in the horizontal and ascending intestinal walls of the duodenum, but no significant enhancement was observed after administration of contrast media. **(b)** High-frequency ultrasound shows the hematoma is located within the intestinal wall, between superior mesenteric artery and the abdominal aorta, presenting as a solid elongated elliptical heterogeneous echo. **(c)** On the 5th day of conservative treatment, abdominal ultrasound re-examination showed an enlarged hematoma in the shape of a sausage, with uneven internal echoes and partial cystic lesions. **(d)** On the 10th day of conservative treatment, high-frequency ultrasound showed a hematoma with a circular shape, mainly cystic inside, and abdominal artery compression. **(e)** On the 15th day of conservative treatment, magnetic resonance imaging showed a circular TI low signal surrounding high signal shadow in the duodenal wall, but no enhancement was observed. **(f)** On the 20th day of conservative treatment, high-frequency ultrasound showed solid hypoechogenicity in the horizontal part of the duodenal wall, with a significant reduction in volume compared to before.

**Figure 2:**
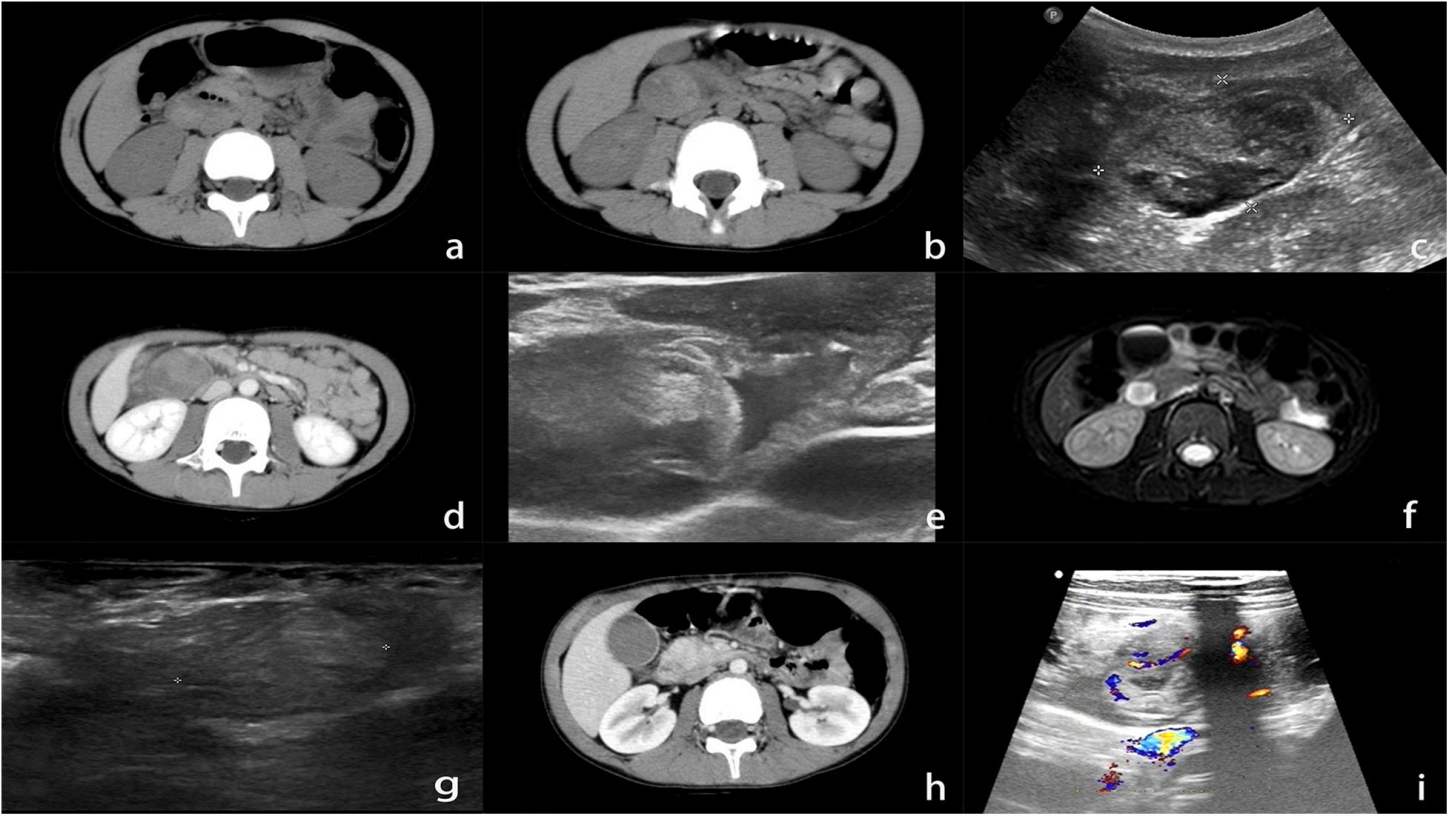
Imaging examination of duodenal wall hematoma in a child with car accident injury. **(a)** About 30 min after the car accident injury, the abdominal computed tomography (CT) scan showed no abnormalities. **(b)** After 10 h, abdominal pain worsened. Re-examination by abdominal CT showed a circular mass shadow in the descending part of the duodenum with uneven density. **(c)** After admission, upper abdominal ultrasound showed a circular heterogeneous echo in the descending part of the duodenum and intestinal wall. **(d)** On the second day of conservative treatment, a CT scan showed that the hematoma in the descending part of the duodenum was slightly larger than before, with uneven density and locally higher density. No enhancement was observed after administration of contrast media. **(e)** On the 5th day of conservative treatment, high-frequency ultrasound showed that the hematoma had reduced in size, accompanied by cystic echoes inside, protruding towards the intestinal cavity. **(f)** On the 10th day of conservative treatment, magnetic resonance imaging re-examination showed a hematoma in the descending wall of the duodenum, presenting as a short TI and long T2 signal shadow. **(g)** On the 12th day of conservative treatment, high-frequency ultrasound showed hematoma shrinkage and low echogenicity. **(h)** On the 15th day of conservative treatment, an abdominal CT scan showed a significant reduction in the hematoma in the descending part of the duodenum. **(i)** On the 20th day of conservative treatment, high-frequency ultrasound showed thickening of the duodenal wall, with hypoechoic hematoma inside. Color Doppler flow imaging showed no blood flow, while blood flow was visible in the surrounding intestinal wall.

All children were re-examined from 3 days after diagnosis to 1 month after discharge. Two patients were re-examined using ultrasound alone: one patient underwent both ultrasound and MRI, one patient underwent both ultrasound and CT, and one underwent ultrasound, CT, and MRI simultaneously. Re-examination results showed that all five cases of hematoma were completely absorbed (Figures 1, 2).

## 4 Discussion

Duodenal intramural hematomas are rare in childhood, accounting for approximately 8.3% of blunt injuries to the duodenum (3). Most children have a clear history of trauma, while a few seek medical attention because of pain, vomiting, or discomfort in the upper abdomen. It is occasionally misdiagnosed as gastritis or duodenal inflammation. Studies have shown that the misdiagnosis and missed diagnosis rate before surgery is as high as 25%–30% (4).

The duodenum is divided into four segments (D1–D4), with the bulb (D1) extending from the pylorus, the ascending segment (D4) being held and fixed by the Qu's ligament, and the descending segment (D2) and horizontal segment (D3) located in the retroperitoneal space without a serosal layer, closely attached to the posterior wall of the abdomen and spine, relatively fixed, and forming a C-shape surrounding the head of the pancreas. The incidence of D2 and D3 injuries was the highest, followed by that of D4 injury. The formation of duodenal wall hematoma is related to its unique anatomy. Due to the wide rib arch margin and relatively wide upper abdomen in children, the abdominal wall muscles are underdeveloped and weak, resulting in insufficient duodenal protection and an increased risk of traumatic hematoma. The duodenum is located in front of the spine and is in a relatively fixed position. It has a dual blood supply within its walls with abundant vascular bundles beneath the serosal and mucosal layers. The anterior and posterior branches as well as the upper and lower branches merge into an arch. When subjected to appropriate external forces, small blood vessels can be easily torn, leading to bleeding. Alternatively, when the upper abdomen is subjected to significant direct external forces, the horizontal segment of the duodenum is prone to shear forces from the posterior spine, causing damage or tearing of the intestinal wall. Duodenal peristalsis is rapid. When subjected to external forces, the pylorus and duodenal jejunal flexure simultaneously close, causing a sudden “closed loop phenomenon” between the pylorus and ligaments, which leads to a sudden increase in pressure in the duodenal cavity, causing blood vessels in the intestinal wall to rupture and bleed, forming a hematoma. When the hematoma grows to push the mucous membrane layer to the opposite side or when a circumferential hematoma causes partial or complete obstruction of the intestinal cavity, it causes duodenal obstruction. After hematoma formation, the mucous membrane layer remains intact and there are no symptoms of peritonitis or internal bleeding. The early clinical manifestations are not specific. When a hematoma grows sufficiently, it manifests as frequent bile vomiting, abdominal pain, and symptoms of discomfort following duodenal obstruction. This explains why children with simple duodenal wall hematomas receive relatively delayed medical treatment. If a child has duodenal ulcers, diverticula, or other lesions, the pressure that the duodenum can withstand will be significantly lower than normal, which is more likely to cause intramural hematoma and even gastrointestinal perforation. There is also the possibility of non-traumatic duodenal hematoma in children with hemorrhagic diseases such as hemophilia and thrombocytopenic purpura, as well as in children undergoing gastroscopy examination (2).

The diagnosis of duodenal interstitial hematoma mainly relies on imaging by abdominal x-ray, CT, MRI, Ultrasound and Contrast-enhanced imaging, etc. Most studies indicate that imaging is the core method for diagnosing traumatic duodenal wall hematomas in children (5). CT and MR have characteristic features: cystic and solid lesions in the upper abdomen closely related to the duodenum, with internal density or signal consistent with changes in hematoma at different stages. Enhanced scanning can more clearly display the relationship, location, and size of bleeding and swelling in the duodenum, providing an important basis for surgical planning and observing therapeutic effects. CT is widely recognized as the gold standard because of its relatively short examination time, high sensitivity to acute bleeding, ability to simultaneously evaluate other abdominal organs, and relatively high equipment penetration rate. However, CT has decreased sensitivity to subacute or chronic hematomas and belongs to the radiation examination category, so it is not recommended for long-term follow-up examinations of pediatric diseases. Although MRI can clearly display the signal characteristics of different stages of hematoma and accurately stage it, it is not as sensitive as CT in detecting the acute phase (<24 h), expensive, time-consuming, and noisy during the examination process, which are not suitable for long-term follow-up use in children. Abdominal x-ray and upper gastrointestinal imaging can only be used as auxiliary examination methods and cannot be used as a qualitative diagnostic basis.

We objectively recognize the limitations of ultrasound technology. Reference (6, 7) points out that ultrasound has low sensitivity and poor accuracy for acute hematoma. This may be due to delayed diagnosis caused by operator experience, dynamic evolution of hematoma, and interference of intestinal gas on cavity organs, which may affect image quality and mask lesions. However, literature (2, 8, 9) clearly emphasizes the irreplaceable cornerstone position of ultrasound in pediatric abdominal trauma assessment. It has strong specificity and characteristic evolution process for duodenal wall hematoma, and also has considerable accuracy in evaluating complications and surrounding organs. This makes it not only suitable for preliminary diagnosis, but also an ideal tool for treatment follow-up due to its nonionizing radiation, noninvasiveness, and reproducibility. As described in reference (10), advanced technologies such as enhanced ultrasound, elastography, ultrafast Doppler, 3D/4D imaging, and high-frequency probes have rapidly developed in recent years. Provide richer diagnostic information from multiple dimensions such as blood flow perfusion, tissue hardness, and microscopic anatomy. These are expected to further enhance the sensitivity and specificity of ultrasound in the diagnosis of organ injury and hematoma staging in the future, and have significant clinical significance in avoiding children from receiving CT radiation multiple times.

Ultrasound examination allows accurate positioning. The duodenal bulb and descending segment surround the pancreatic head in a C-shape on the right side, which makes it easier to locate. A hematoma in this segment was confirmed by observing its relationship with the pancreas. The position of the horizontal part is relatively fixed, and the transverse sections of the SMA and AO are found in the upper abdomen. Between them is the horizontal part of the duodenum, which is the only intestinal tract in this area. The hematoma in the horizontal segment was precisely sandwiched between the two, with the SMA elevated and the AO compressed, making it easier to determine this lesion. The horizontal part passing through the left side of the angle between the SMA and AO is the ascending part. High-frequency ultrasonography can clearly display the structure of each layer of the duodenal wall, accurately locate the location of the hematoma, and distinguish between the mucosal, muscular, and serosal layers. Simultaneously, it can clearly display the condition inside the intestinal cavity, observe intestinal peristalsis in real time, evaluate the degree of intestinal obstruction caused by a hematoma, and allow adjustment of the treatment plan in real time. In this study, high-frequency ultrasound was used to accurately locate the hematoma in all five cases, and intestinal obstruction was evaluated multiple times.

Ultrasound examination allows disease assessment. The hematoma presents as an oval or sausage-shaped mass without peristalsis. Over time, the trend of echo change was heterogeneous echo, no echo, and low echo. This change may be caused by the decomposition and absorption of blood components in the hematoma over time, resulting in fibrosis or the formation of non-absorbable gel-like substances. We identified three cases with this manifestation, two of which showed low echogenicity owing to small hematomas. Ultrasound examination also allows identification of secondary lesions. A hematoma in the descending segment of the duodenum can compress the greater papilla of the duodenum, leading to dilation of the bile and pancreatic ducts. One patient experienced dilation of the pancreatic and common bile ducts, as well as enlargement of the gallbladder. Duodenal hematomas generally occur between the submucosal and muscular layers, pushing the muscular and mucosal layers, and causing narrowing and obstruction of the intestinal lumen. All five patients in this study had varying degrees of secondary intestinal obstruction. One patient had a hematoma located between the muscular and serosal layers; however, the hematoma was large, and the compression symptoms were obvious.

Several potential differential diagnoses should be considered. Duodenal wall hematoma should be distinguished from common intestinal wall adenomas, leiomyomas, and malignant tumors. Intestinal wall adenomas often occur in the bulbous and descending parts, with slightly strong echogenicity on ultrasound, a diameter generally approximately 10 mm, and protrude into the intestinal lumen. Smooth muscle tumors of the intestinal wall are generally slightly larger, appearing as homogeneous hypoechoic masses on ultrasound with mild-to-moderate enhancement on CT, and are easily distinguishable from non-enhanced intramural hematomas. Malignant tumors of the intestinal wall often present with uneven thickening of the local intestinal wall, interruption of the mucosal layer, unclear boundaries, infiltration and adhesion of surrounding soft tissues, invasion of fat spaces, and distant metastasis. Duodenal lymphoma is often characterized by uniform thickening of the intestinal wall, and dilation of the intestinal lumen. Pancreatic hematomas cannot be effectively distinguished owing to their proximity to the duodenum. However, with the widespread application of high-frequency ultrasound, the precise localization of abdominal diseases has become increasingly advanced, and the accuracy and specificity of disease diagnosis has improved.

Based on the above, this article proposes that for children considering duodenal wall hematoma, immediate emergency upper abdominal CT enhancement examination should be performed (there may be cases where the onset time is too short, the rupture of small blood vessels in the mucosa and serosa has not yet formed a significant hematoma and requires several hours or reexamination after obvious symptoms). After diagnosis, ultrasound follow-up examinations can be conducted multiple times. For experienced ultrasound physicians, based on medical history and relevant signs, they can accurately locate hematomas and observe their changes, providing clinical treatment guidance. For young ultrasound doctors, by combining CT images and carefully examining them, they can also accurately locate hematomas.

The treatment plan was relatively clear after the diagnosis of the duodenal intramural hematoma. Conservative treatment is usually the primary approach and involves continuous gastrointestinal decompression and total intravenous nutritional support. Multiple studies (11, 12) have shown that conservative success rate is 88%, supporting nonsurgical options as the preferred option. However, there is currently no consensus on the timing. Early literature advocated conservative treatment before surgery, but there is no clear record of appropriate or maximum time limits. Some authors (13) believe that once diagnosed and treated for a week without improvement in symptoms, immediate surgical treatment should be performed. The reason is that surgical can shorten the course of the disease, and at the same time, the time for hematoma absorption in each child is difficult to determine. Early surgical methods are simple, postoperative recovery is fast, and intestinal stenosis caused by hematoma absorption organization is avoided, which would increase the complexity of subsequent surgery. But more experts (1, 14) believe that for diagnosed simple duodenal intramural hematoma, the principle of conservative treatment should be adhered to, and premature surgery should not be performed due to unclear short-term effects. Although it can shorten the course of the disease, it leaves behind permanent incision scars and the risk of adhesive intestinal obstruction. However, for those whose condition has not improved after conservative treatment for 3–4 weeks or more, surgical treatment is still recommended (12).

Traumatic intramural duodenal hematomas are rare in children, and conservative treatment such as gastrointestinal decompression and intravenous nutrition has a good prognosis. Initial CT diagnosis combined with ultrasound multiple follow-up can timely and accurately diagnose diseases, and multiple follow-up examinations can provide strong basis for clinical decision-making, reduce unnecessary surgeries, and shorten the hospitalization time of children.

## Data Availability

The original contributions presented in the study are included in the article/Supplementary Material, further inquiries can be directed to the corresponding author.
